# Application of Antibody Fragments Against Aβ With Emphasis on Combined Application With Nanoparticles in Alzheimer’s Disease

**DOI:** 10.3389/fphar.2021.654611

**Published:** 2021-04-22

**Authors:** Zhi-Ting Sun, Chi Ma, Guang-Jian Li, Xiang-Yu Zheng, Yi-Tong Hao, Yu Yang, Xu Wang

**Affiliations:** ^1^ Department of Neurology and Neuroscience Center, The First Hospital of Jilin University, Changchun, China; ^2^ Department of Neurosurgery, The First Hospital of Jilin University, Changchun, China

**Keywords:** antibody fragments, amyloid-β, nanoparticle, Alzheimer's disease, immunotherapy

## Abstract

Alzheimer’s disease (AD) is one of the most common neurodegenerative diseases and accumulating evidences suggest a key role of amyloid-β (Aβ) peptide in the pathogenesis of AD. According to the amyloid cascade hypothesis, the imbalance of producing and clearing Aβ is the beginning of neurodegeneration and dementia. Consequently, immunotherapy becomes popular through using antibodies against Aβ. However, many studies of monoclonal antibodies were stopped because adverse effects appeared or there were no evident benefits observed. Some antibody fragments have many advantages over monoclonal antibodies, such as small sizes, lack of the crystallizable fraction (Fc) and so on. There are three main antibody fragments, including single chain variable fragments (scFvs), Fab fragments and single-domain antibody fragments. Nanoparticles can facilitate the entry of drug molecules across the blood-brain barrier, making them become excellent carriers. Various kinds of nanoparticles have been applied in the treatment of AD. The combination of nanoparticles and antibody fragments against amyloid-β can be used in the diagnosis and treatment of Alzheimer’s disease. In this review, we summarize the progress of antibody fragments against amyloid-β in AD, focusing on the combined application with nanoparticles in the diagnosis and treatment of AD.

## Introduction

Alzheimer’s disease (AD) is the most common cause of dementia (Holmes and Amin, 2020). One of the important pathological characteristics of AD is the extracellular aggregation of amyloid plaques, mostly consisted of amyloid-β (Aβ) peptide. (Uddin et al., 2020). According to the amyloid cascade hypothesis, Aβ plays a key role in the development of AD (Uddin et al., 2020). Aβ is accumulated because it is overproduced or there is deficiency in elimination (Uddin et al., 2020). The excess of Aβ results in the aggregated fibrils and neurotoxic oligomers. Immunotherapy targeting Aβ to promote the elimination of Aβ has become a promising strategy to treat AD (Behl et al., 2020). Bapineuzumab is a monoclonal antibody (mAb) against Aβ, which was terminated in phase 3 clinical trials (Loureiro et al., 2020). Many second generation of anti-amyloid mAbs have been studied and undergone clinical trials. However, lots of clinical trials were terminated because results were not successful (Tian Hui Kwan et al., 2020). These monoclonal antibodies that entered phase 3 clinical trials mainly include Crenezumab, Solanezumab, Gantenerumab and Aducanumab (Tian Hui Kwan et al., 2020). These studies of Solanezumab and Crenezumab were terminated due to lack of efficacy. In terms of Gantenerumab and Aducanumab, although many studies were also terminated, there are researches still in progress (Tian Hui Kwan et al., 2020).

Some antibody fragments preserve the ability of combining with antigen, which can replace complete mAbs (Bitencourt et al., 2020). The size of antibody fragment is small, enhancing the capacity of passing through BBB. Besides, they can be very useful in imaging and the manufacturing cost is not high. There are three main kinds of antibody fragments, including single chain variable fragment (scFv), Fab fragment and single-domain antibody fragment (Bitencourt et al., 2020). Applying antibody fragments and combining them with nanoparticles in the diagnosis and treatment of AD are mainly discussed in this review. These antibody fragments against different epitopes of Aβ in AD are summarized in Table 1. And the antibody fragments against Aβ oligomers are summarized in Table 2 **.**

**TABLE 1:** Antibody fragments against different epitopes of Aβ in AD.

Epitope	Antibody fragments	Investigation model	Comments	Reference
N-terminal region of Aβ	ScFv-h3D6	SH-SY5Y cells	Inhibit amyloid fibril formation and cytotoxicity, improve memory and learning abilities, decrease levels of apoE, apoJ and tau, reduce levels of Aβ oligomers and IL-6, increase volume of brain and keep neurons within DCN from death, target oligomers, monomers, fibrils	Marin-Argany et al. (2011); Esquerda-Canals et al. (2013); Gimenez-Llort et al. (2013); Roda et al. (2020b); Guell-Bosch et al. (2020)
		3xTg-AD mice		
	ScFv-IC16	7PA2 cells	As a probe to detect Aβ aggregation, target monomers, oligomers, fibrils	Dornieden et al. (2013)
		Tg2576 mice		
	ScFv HT7	SH-SY5Y cells, HUVEC and C6 glioma cells	Prevent aggregation of Aβ, decrease cytotoxicity and transport the BBB with high efficiency, target oligomers, protofibrils, fibrils	Zhang Y. et al. (2019)
	ScFv9	*Drosophila*	Improve memory impairment significantly	Martin-Pena et al. (2017)
	A8 scFv	A model of cell-free Aβ “on-pathway” aggregation	Prevent aggregation of Aβ and disaggregate fibrils	Zhang et al. (2015c)
	WO-2 Fab	neuroblastoma cells	Inhibit the toxicity caused by Aβ oligomer and aggregation, boost the disaggregation of amyloid fibrils	Robert et al. (2010)
	F(ab)’2 fragment of an IgG1 mAb	Tg2576 mice	Decrease Aβ plaque formation	Tamura et al. (2005)
	Fab fragment of NT4X	Transgenic mice (Tg4-42 mice and 5XFAD mice)	Therapeutic effect on loss of neurons and memory impairment	(Antonios et al., 2015)
Central region of Aβ	B4.4	SH-SY5Y cells	Neutralize toxicity of Aβ	Solorzano-Vargas et al. (2008)
	H1v2 scFv	SH-SY5Y cells	Reduce Aβ aggregation and eliminate toxicity	Liu et al. (2004)
	1E8 scFv	PC12 cell line and murine primary neurons	Decrease formation of fibril and protect cells from toxicity	Nisbet et al. (2013)
	ScFv17	APP/PS1 transgenic mice	Decrease pathological impairments	Hu et al. (2018)
C-terminal region of Aβ	VHH V31-1	Amyloids from AD patient brain tissue, SK-N-SH cells	Prevent toxicity and formation of fibrils	Lafaye et al. (2009)
	ScFv42.2	*Drosophila*	Improve memory deficit significantly	Martin-Pena et al. (2017)

BBB, blood-brain barrier; DCN, deep cerebellar nuclei; ScFv, single chain variable fragment.

**TABLE 2:** Antibody fragments against Aβ oligomers.

Antibody fragments	Investigation model	Comments	Reference
W8	SH-SY5Y cells	Inhibit fibrillation of Aβ and prevent cytotoxicity	Wang X.-p. et al. (2009)
W9			
W20			
WC2			
A4 scFv	SH-SY5Y cells	Inhibit Aβ aggregation and decrease the toxicity	Zameer et al. (2008)
	Brain tissues of PD, AD and non disease patients		
C6	7PA2 cells (a CHO cell line that over-expresses hAPP) wild type and triple transgenic (3xTg) mice	Combine with oligomeric Aβ specifically	Kasturirangan et al. (2013)
NUsc1	Hippocampal neurons	Comebine with Aβ oligomers with high specificity, decrease oxidative stress of neuron, and decrease tau hyperphosphorylation	Sebollela et al. (2017)
	AD-transgenic mice and wild type mice		
MO6	SH-SY5Y cells, HUVEC and C6 glioma cells	Cross the BBB, reduce cytotoxicity, and increase cell viability	Zhang et al. (2015a)
AS	SH-SY5Y cells, HUVEC and C6 glioma cells, rat primary neurons	Pass through the BBB, prevent cytotoxicity, and decrease the level of Aβ	Zhang et al. (2015b)
ScFv-IC16	7PA2 cells	As a probe to detect Aβ, inhibit Aβ aggregation	Dornieden et al. (2013)
	Tg2576 mice		
ScFv HT7	SH-SY5Y cells, HUVEC and C6 glioma cells	Prevent aggregation of Aβ, decrease cytotoxicity and transport the BBB with high efficiency	Zhang Y. et al. (2019)
scFv HT6	SH-SY5Y cells	Prevent Aβ aggregation and decrease cytotoxity	Zhang X. et al. (2019)
10D5-scFv	Pheochromocytoma (PC12) cells	Prevent from forming oligomers and fibers of Aβ and inhibit toxicity	Fu et al. (2020)
12B4-scFv	APPswe/PS1dE9 transgenic mice		
ScFv-h3D6	SH-SY5Y cells	Inhibit formation of amyloid fibril, and protect cells from toxicity, improve memory and learning abilities, decrease levels of apoE, apoJ and tau, reduce levels of Aβ oligomers and IL-6, increase volume of brain and keep neurons within DCN from death	Marin-Argany et al. (2011); Esquerda-Canals et al. (2013); Gimenez-Llort et al. (2013); Roda et al. (2020b); Guell-Bosch et al. (2020)
	3xTg-AD mice		
VHH V31-1	Amyloids from AD patient brain tissue, SK-N-SH cells	Prevent toxicity and formation of fibrils	Lafaye et al. (2009)

BBB, blood-brain barrier; DCN, deep cerebellar nuclei; ScFv, single chain variable fragment.

## The Application of Antibody Fragments Against Aβ in AD

### The Application of scFvs Against Aβ in AD

Applying scFv is safer compared with using compete mAb, because scFv do not contain the crystallizable fraction (Fc), which activates microglia and triggers complement system (Bitencourt et al., 2020). Therefore, Fc fragment is related to adverse effects produced by mAb. These adverse effects were part of the reason of stopping the clinical trials of Bapineuzumab (Bitencourt et al., 2020). Another reason was that researchers did not obtain treatment effect. The beginning of the treatment with Bapineuzumab was late in the clinical trials, which had been considered to be a reason for not obtaining clinical benefits. Therefore, treating AD in a prodromal stage is necessary (Tian Hui Kwan et al., 2020).

These scFvs can target different linear epitopes of Aβ, including N-terminal region, central region and C-terminal region. Besides, they can also target various conformational epitopes, which contain monomers, oligomers, protofibrils and fibrils (Robert and Wark, 2012).

#### ScFvs Targeting the N-Terminal Region of Aβ (Amino Acids 1-16)

##### Experiments of scFvs Targeting the N-Terminal Region of Aβ *In Vitro*

ScFv-h3D6 was obtained from Bapineuzumab (Esquerda-Canals et al., 2019b). It was proved to be able to inhibit cytotoxicity caused by Aβ peptide in the SH-SY5Y neuroblastoma cell line. The formation of amyloid fibril was also prevented by it (Marin-Argany et al., 2011). The conformational mechanism was relevant to the worm-like pathway (Marin-Argany et al., 2011). As for producing scFv-h3D6, there are many problems in producing it from *Escherichia coli*, such as the existence of endotoxins. Therefore, producing it from *Pichia pastoris* was studied by Montoliu-Gaya et al. (2017a). In terms of inhibition of toxicity, they proved that effects of scFv-h3D6 were not changed whether it was obtained from *Escherichia coli* or from *Pichia pastoris* (Montoliu-Gaya et al., 2017a). The effect of inhibiting toxicity caused by Aβ was evaluated in the SH-SY5Y neuroblastoma cell line. Besides, obtaining this antibody fragment from *Pichia pastoris* had more advantages than from *Escherichia coli*, which made *Pichia pastoris* a better choice for producing scFv-h3D6 (Montoliu-Gaya et al., 2017a). Montoliu-Gaya et al. (2017b) also found that the production yield could be increased by eliminating the disulfide bond of the V_H_ domain, resulting in the absence of scrambling conformations (Montoliu-Gaya et al., 2017b).

ScFv-IC16 could identify different Aβ species, including monomers, oligmers and protofibrils, which was confirmed by ELISA analysis (Dornieden et al., 2013). And scFv-IC16 was able to stain Aβ plaques in the brain slices of AD transgenic mice by immunohistochemistry. Therefore, scFv-IC16 could be used as a molecular probe of detecting Aβ, which was potential for diagnosing and treating AD (Dornieden et al., 2013). A8 scFv, expressed in baculovirus, also could prevent the aggregation of Aβ in a model of cell-free Aβ aggregation (Zhang et al., 2015c). Besides, HT7 was obtained from the scFv antibody library of human, which was contributed by a healthy donor (Zhang Y. et al., 2019). HT7 antibody could disaggregate the Aβ42 aggregates *in vitro* and inhibit cytotoxicity caused by Aβ42 in SH-SY5Y cells. The mechanism of Aβ42 oligomeric subunits for effective anti-Aβ42 antibodies called "post-saturation dissociation" was raised (Zhang Y. et al., 2019).

##### Experiments of scFvs Targeting the N-Terminal Region of Aβ *In Vivo*

Gimenez-Llort et al. (2013) studied the protective effects of scFv-h3D6 in the 3xTg-AD mice. They found that scFv-h3D6 could be effective in many aspects of 3xTg-AD mice, such as the recovery of swimming speed, improvement of memory and enhancement of learning ability. Treatment with scFv-h3D6 reduced the level of Aβ oligomers and apolipoproteins, including apoE and apoJ (Gimenez-Llort et al., 2013). Besides, Esquerda-Canals et al. (2013) also studied functions of scFv-h3D6 in the 3xTg-AD mice. They focused on deep cerebellar nuclei (DCN) neurons. In the early phase of AD, some of these neurons could be dead. The scFv-h3D6 fragment could protect neurons from death, but not all of them, because of the insufficient dose in this study (Esquerda-Canals et al., 2013). Afterward, Esquerda-Canals et al. (2019b) observed that levels of intracellular Aβ were decreased in the 3xTg-AD mice after injecting scFv-h3D6. It could prevent neurons from death. They also found that cognitive function was improved in terms of spatial memory according to the Morris Water Maze (MWM) tests (Esquerda-Canals et al., 2019b). Moreover, the treatment did not cause neuroinflammation. It was not harmful to the functions of kidney and liver, either. However, there were influences on spleen shown during the treatment, which indicated that spleen might be related to clearance of the combination of Aβ and scFv-h3D6 (Esquerda-Canals et al., 2019b). Roda et al. (2020a) proved the effect of scFv-h3D6 on decreasing level of Aβ and improving impairment of cognition in the 3xTg-AD mice (Roda et al., 2020a). They suggested that applying scFv-h3D6 and stimulation of cognition together might be a strategy for treating AD (Roda et al., 2020a). Moreover, they found that levels of Aβ and tau in the 3xTg-AD mice were both decreased after applying it. They also showed that scFv-h3D6 did not induce the response of inflammation (Roda et al., 2020b). Furthermore, another study also described the therapeutic effects of scFv-h3D6 in the 3xTg-AD mice. The volume of brain was increased while levels of IL-6 and Aβ were decreased after applying scFv-h3D6 (Guell-Bosch et al., 2020). In terms of producing scFv-h3D6, it was proved that scFv-h3D6 could decrease the level of Aβ in the 3xTg-AD mice whether it was obtained from *Escherichia coli* or from *Pichia pastoris* (Montoliu-Gaya et al., 2017a). And scFv9 could protect effectively against memory deficit of *drosophila* caused by Aβ42 deposits (Martin-Pena et al., 2017).

#### ScFvs Targeting the Central Region of Aβ (Amino Acids 17-32)

##### Experiments of scFvs Targeting the Central Region of Aβ *In Vitro*

B4.4 could combine with the central part of Aβ_1-42_ and inhibit the toxicity of fibrillar Aβ_1-42_ and oligomeric Aβ_1-42_ in SH-SY5Y cells (Solorzano-Vargas et al., 2008). Some scFvs have effects on inhibiting aggregation of Aβ. Liu et al. (2004) discovered that H1v2 could combine with the 17-28 region of Aβ and have effects on inhibiting the aggregation of Aβ. It could also eliminate the toxic effects caused by aggregation of Aβ in SH-SY5Y cells (Liu et al., 2004). Besides, 1E8 scFv was obtained successfully and could combine with the central part of Aβ (Nisbet et al., 2013). It was proven that 1E8 scFv could decrease fibril formation of Aβ_1-42_. The toxicity caused by Aβ_1-42_ could be inhibited by 1E8 scFv in murine primary neuronal cultures and the P12 cell line (Nisbet et al., 2013). Besides, scFv 4.8 and scFv 3.20 prevented toxicity caused by oligomeric Aβ_1-42_ in neuroblastoma cell cultures (Medecigo et al., 2010). The 25-35 fragment of Aβ promotes Aβ aggregation and causes toxicity. The study showed that scFvs called B6 and D4 against 25-35 fragment of Aβ could restrain the aggregation of Aβ42 and decrease the toxicity caused by Aβ42 in SH-SY5Y cells (Zameer et al., 2006).

##### Experiments of scFvs Targeting the Central Region of Aβ *In Vivo*

There were five fragments chosen and studied by Medecigo et al. (2010). ScFv 4.8 and scFv 3.20 could target central region of Aβ. After the intracranial injection, it was observed that scFv 4.8 and scFv 3.20 identified amyloid deposits and decreased amyloid deposits specifically in the brains of APP/Tg2576 mice (Medecigo et al., 2010).

#### ScFvs Targeting the C-Terminal Region of Aβ (Amino Acids 33-42)

ScFv42.2 targeted the C-terminal region of Aβ. It could protect against memory deficit of *drosophila* (Martin-Pena et al., 2017). *Drosophila* model of AD can be used in studying the neuroprotective effect of novel scFvs. It was shown that scFv42.2 could inhibit the loss of neurons and improve neuron function. The effect of applying scFv9 and scFv42.2 together was also studied in *drosophila* (Fernandez-Funez et al., 2015). It was proven that their protective functions were synergistic, which indicated that applying scFvs targeting different epitopes together might be a more effective way to treat AD (Fernandez-Funez et al., 2015). ScFv17 targeting Aβ31-35 was obtained through genetic engineering technology of phage display (Hu et al., 2018). It was proven that scFv17 could penetrate BBB easily and had evident effects on decreasing the levels of Aβ oligomers and Aβ plaques in APP/PS1 transgenic mice (Hu et al., 2018).

#### ScFvs Targeting a Conformational Epitope (Monomers, Oligomers, Protofibrils and Fibrils)

More and more evidences have shown that Aβ oligomers, instead of fibrils or monomers, is the main toxic form inhibiting synaptic plasticity (Wang X.-p. et al., 2009). According to the study, four scFv antibodies including W8, W9, W20 and WC2, were obtained from human scFv library through phage display, which identified Aβ oligomers specifically (Wang X.-p. et al., 2009). All of these scFv antibodies could combine with Aβ oligomers and prevent against the cytotoxicity in SH-SY5Y cells and fibrillation of Aβ (Wang X.-p. et al., 2009). A4 scFv antibody targeting Aβ oligomers was proven to restrain Aβ aggregation and decrease the toxicity in SH-SY5Y cells (Zameer et al., 2008). Besides, A4 scFv was able to combine with Aβ aggregates in brain tissues of AD patients (Zameer et al., 2008). C6 scFv can also combine with oligomeric Aβ in 7PA2 cells and brain tissues of triple transgenic mice (Kasturirangan et al., 2013). It could be helpful in diagnosing neurodegenerative diseases and evaluating the treatment and development of disease (Kasturirangan et al., 2013). Similar with C6, NUsc1 is a scFv which targets Aβ oligomers (Sebollela et al., 2017). It is potential to be used in the diagnosis and treatment in AD (Sebollela et al., 2017). It was reported that scFv MO6 could identify and combine with the oligomeric Aβ42 selectively (Zhang et al., 2015a). It could decrease levels of oligomeric Aβ42 by preventing their formation and disaggregate Aβ42 aggregation. ScFv MO6 could also reduce the cytotoxicity caused by Aβ42 and improve viability of cell. And scFv MO6 could cross the BBB model *in vitro* (Zhang et al., 2015a). Moreover, scFv AS was identified and could combine with immature protofibrils as well as medium-size Aβ oligomers (Zhang et al., 2015b). ScFv AS could decrease levels of Aβ oligomers through preventing their formation or leading to their disaggregation (Zhang et al., 2015b). In this way, Aβ cytotoxicity was inhibited. ScFv AS could also transport across the BBB model *in vitro* (Zhang et al., 2015b). What is more, scFv HT6 was able to combine with medium-sized Aβ42 aggregation and lead to disaggregation of large Aβ42 aggregation into small Aβ42 oligomers (Zhang X. et al., 2019). At the same time, it could inhibit the progress of Aβ42 aggregation. The effect of scFv HT6 on decreasing the cytotoxicity caused by Aβ42 oligomers was evident (Zhang X. et al., 2019). HT7 could target oligomers, protofibrils and fibrils (Zhang Y. et al., 2019). Besides, scFv-IC16 could recognize monomers, oligomers and fibrils (Dornieden et al., 2013). Besides, there are many scFvs targeting several conformational epitopes. Both 12B4-scFv and 10D5-scFv could combine with Aβ fibers, monomers and oligomers, as well as Aβ plaque successfully. Moreover, the study showed that 10D5-scFv could combine with Aβ fibers and oligomers better than 12B4-scFv (Fu et al., 2020).

#### Adeno-Associated Virus as Vectors of scFvs

Adeno-associated virus (AAV) can be vectors of scFvs (Levites et al., 2006). Levites et al. (2006) found that Aβ deposition was reduced after injecting AAV1 containing the scFv (scFv9, scFv40.1 and scFv42.2) into the ventricles of CRND8-transgenic mice. Another study about injecting AAV containing the scFv59 into the corticohippocampal parts showed that the amyloid deposits were decreased compared with that of mice injected with PBS (Fukuchi et al., 2006). The existence of scFv59 could be detected easily in the neurons in a year after injecting AAV containing the scFv59 (Fukuchi et al., 2006). However, immunotherapy through recombinant AAV (rAAV) can cause hemorrhage by injecting it into ventricle (Kou et al., 2011). Researchers should pay attention to this point (Kou et al., 2011). Delivering scFv59 directed by muscle through rAAV1 was proven to be an efficient way of reducing Aβ deposits in the brain (Yang et al., 2013). Reduced levels of Aβ were observed in cerebrospinal fluid (CSF). However, incremental levels of Aβ were shown in the serum (Yang et al., 2013). Wang Y.-J. et al. (2009) proved that delivering the scFv gene through AAV in an intramuscular way or in an intracranial way did not cause the increase of microhemorrhage, activation of microglia or evocation of T cells. Besides, the effect was similar in these two ways. The level of Aβ was increased in serum while was decreased in the brain (Wang Y.-J. et al., 2009). The effect of delivering scFv gene intramuscularly was significant on inhibiting the formation of Aβ plaque and preventing the accumulation of Aβ. Besides, the cognitive impairment was also ameliorated through delivering scFv gene intramuscularly. Wang et al. (2010) proved that delivering scFv gene intramuscularly did not induce microhemorrhage or inflammation in the brain of APP_Swe_/PS1dE9 transgenic mice (Wang et al., 2010). After intrahippocampally injecting rAAV1, insoluble Aβ was decreased and Morris Water Maze (MWM) showed that the cognition of mice was improved (Ryan et al., 2010).

### The Application of Fab Fragments in AD

#### Experiments of the Application of Fab Fragments in AD *In Vitro*

It was proven that 1E8-4b Fab could identify Aβ [1-40/42/43] peptides (Tammer et al., 2002). 1E8-4b Fab was able to combine with plaques in the brain tissue sections from CERAD-defined AD patients by immunohistochemistry (Tammer et al., 2002). It was demonstrated that rFab of WO-2 had the ability of restraining the neurotoxicity caused by Aβ_1-42_ oligomer and depolymerizing the Aβ_1-42_ fibrils formed previously (Robert et al., 2009). It was shown that humanized WO-2 Fab had the ability of inhibiting the toxicity caused by Aβ oligomer and aggregation (Robert et al., 2010). It could boost the disaggregation of amyloid fibrils (Robert et al., 2010). Fab can be combined with a quenchbody (Q-body), which is an antibody-based biosensor. This combination can be used in detection and imaging of Aβ aggregation, which may help diagnose AD (Dong et al., 2018).

#### Experiments of the Application of Fab Fragments in AD *In Vivo*

4396C is a mAb targeting GM1 ganglioside-bound Aβ (GAβ) specifically (Yamamoto et al., 2005). According to a study, Fab fragments of 4396C were effective in the inhibition of Aβ deposition in transgenic mice after the intraperitoneal injection of Fab fragments (Yamamoto et al., 2005). F(ab)’2 fragment belongs to an IgG1 mAb. The F(ab)’2 fragment was able to decrease Aβ plaque formation in Tg2576 mice after the injection of it, whether in an intracranial way or in an intraperitoneal way (Tamura et al., 2005). Application of the F(ab)’2 fragment decreased the infiltration of phagocytes compared with the complete mAb (Tamura et al., 2005). The Fab fragment which belonged to NT4X had the therapeutic effect on loss of neurons and memory impairment (Antonios et al., 2015). 

### The Application of Single-Domain Antibody Fragments in AD

#### Experiments of the Application of Single-Domain Antibody Fragments in AD *In Vitro*

The VHH is a single-chain domain binding to the antigen, which lacks light chain. The recombinant VHH has an intact antigen-binding domain with minimal size (Lafaye et al., 2009). VHHs acquired from an immunized alpaca phage display library, could identify Aβ oligomers with low molecular-weight (MW) selectively in neurons (Lafaye et al., 2009). VHH V31-1 was able to inhibit the formation of fibril and prevent the neurotoxicity caused by Aβ (Lafaye et al., 2009). The study suggested that VHH V31-1 was potential in imaging and diagnosing AD (Lafaye et al., 2009). Hydrophobic complementarity-determining regions (CDRs) are able to boost the aggregation of antibody (Perchiacca et al., 2012). It was observed that the solubility of VHH could be increased by changing the sequence of CDR loops within them. In this way, the aggregation of antibodies would be resisted while their binding affinity would not be decreased. VHH used in this study was specific for Aβ peptide and their specificity did not change (Perchiacca et al., 2012). Gammabody is a VH antibody against Aβ, which could identify Aβ aggregations specifically with high affinity and decrease Aβ toxicity (Zhang et al., 2018). VH antibody fragments obtained from the mouse immune library could decrease the toxicity caused by oligomeric Aβ42 (Medecigo et al., 2010). The specific combination of VH antibody fragments and Aβ deposits was observed in the brain of Tg2576 mouse (Medecigo et al., 2010).

#### Experiments of the Application of Single-Domain Antibody Fragments in AD *In Vivo*

The size of llama VHH is small, which is about 12–15 kDa (Rotman et al., 2015b). It could cross the BBB, but the BBB passage was poor (Rotman et al., 2015b). It was observed that VHHs could extravagate slowly to pass the BBB. It was shown that VHHs diffused in the parenchyma. Besides, the tangles of neurofibril and deposits of amyloid were labeled by VHHs (Li et al., 2016). After delivering a VH fragment in an intracranial way, this fragment could decrease the amyloid burden in the Tg2576 mouse (Medecigo et al., 2010).

## The Combined Application of Antibody Fragments and Nanoparticles in AD

**FIGURE 1:**
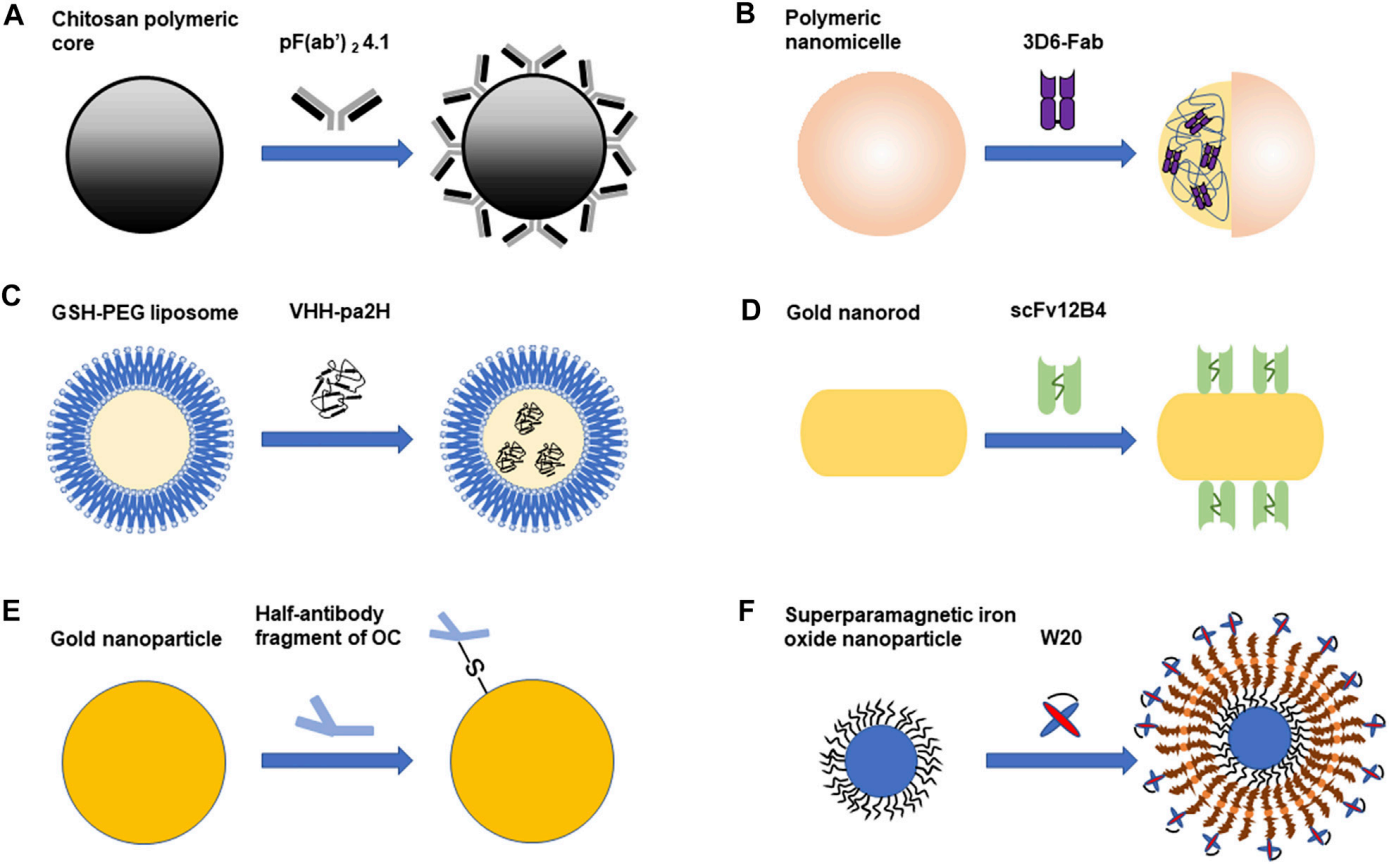
The combined application of antibody fragments and nanoparticles in Alzheimer’s disease. **(A)**. PF(ab')_2_ 4.1 cover the surface of the chitosan polymeric core. **(B)**. Polymeric nanomicelle encapsulates 3D6-Fab. **(C)**. GSH-PEG liposome encapsulates VHH-pa2H **(D)**. ScFv 12B4 are combined with gold nanorod (GNR). **(E)**. Half-antibody fragment of OC is combined with gold nanoparticle (AuNP). **(F)**. W20 is combined with superparamagnetic iron oxide nanoparticle (SPION). Different antibody fragments combine with different nanoparticles. Some antibody fragments are on the surface of the nanoparticle, while some are encapsulated in the nanoparticle. The combination of antibody fragments and nanoparticles can be used in the diagnosis or treatment of Alzheimer’s disease.

Nanoparticles can serve as drug delivery carriers to cross the BBB (Thangudu et al., 2020). Nanoparticles have many advantages. For example, nanoparticles can make hydrophobic drugs more stable and effective. And they can help drugs to target at tissues specifically and increase efficacy. Therefore, less doses can be effective and adverse effects will be decreased (Thangudu et al., 2020). Nanoparticles can pass through the BBB because their small sizes, often between 1 and 100 nm (Thangudu et al., 2020). Different kinds of nanoparticles have been used in the diagnosis and treatment of AD, including polymeric nanoparticles, liposomes, magnetic nanoparticles and etc (Liu et al., 2019). Nowadays, the combination of nanoparticles and antibody fragments has been applied in the diagnosis and treatment of Alzheimer’s disease. The following studies related to the combined application of antibody fragments and nanoparticles are summarized in Figure 1 **.**

### The Combination of Fab Fragments and Polymeric Nanoparticles

Polymeric nanoparticles are promising to be used in diagnosis and therapy of AD if they can pass through the BBB efficiently. Chitosan nanoparticles belong to polymeric nanoparticles (Agyare et al., 2008). There are several advantages of the chitosan nanoparticles (Agyare et al., 2008): 1) no allergic reactions induced; 2) effects on improving absorption of drugs; 3) The producing condition is very mild, which guarantee the completeness of proteins and etc.; 4) the degradation product of chitosan in the body is nontoxic. However, it was still challenging for these nanoparticles to target amyloid deposits in the brain specifically (Agyare et al., 2008). In order to solve this problem, smart nano-vehicles (SNVs) were designed by Agyare et al. (2008). The polymeric core of SNV was made from chitosan nanoparticle. The pF(ab′)_2_ was used as the biosensor, which covered the surface of the nanoparticle core. PF(ab′)_2_ could detect deposits of amyloid specifically. PF(ab′)_2_ was obtained by using putrescine to modify a F(ab')_2_ fragment. It belongs to IgG4.1 against Aβ. The pF (ab′)_2_ could cross BBB and combine with plaques and deposits of amyloid. Therefore, SNVs in this study were the chitosan polymeric cores (CPCs) covered with PF(ab′)_2_. Meanwhile, CPCs covered with a bovine serum albumin (BSA) were made as a control nano-vehicle (CNV). BSA applied in the control group was not specific and its molecular weight was similar to pF (ab′)_2_. The study showed that uptake of ^125^I-SNVs was much more than that of ^125^I-CNVs in the brain of mice. Therefore, they suggested that pF(ab′)_2_ enhanced the transcytosis of SNVs in the endothelial cells of BBB (Agyare et al., 2008). Afterward, Agyare et al. (2014) had another study about combining chitosan with pF(ab′)_2_. Different with the previous system, they added a MRI contrast agent and cyclophosphamide (CYC). This new system was called theranostic nanovehicles (TNVs). The MRI contrast agent Magnevist® could detect the early stage of cerebral amyloid angiopathy (CAA). They loaded the core with CYC, which was able to inhibit cerebrovascular inflammation. They found that the productions of pro-inflammatory cytokine were decreased more effectively by using TVNs than using CYC alone. PF(ab′)_2_4.1 that was on the surface of this nanocore could combine with amyloid. TVNs were able to target accumulation of amyloid in the brain of mice. Besides, they observed that TVNs could prevent production of cytokines caused by the exposure of Aβ40 (Agyare et al., 2014). Another application of combining polymeric nanoparticles and antibody fragment is studied by Xie et al. (2020). The system of polymeric nanomicelle (PM) was provided to deliver 3D6-Fab. After applying the system of PM, they found that 3D6-Fab was transported more into the brain of AD mice than using this fragment alone. The accumulation of this fragment was 41 times in the system of PM than using 3D6-Fab without this system. It was observed the aggregation of Aβ_1-42_ was restrained in the way of transporting 3D6-Fab through this system. 3D6-Fab absorbed by peripheral tissues was minimal. It was proven that the effect on treating AD was increased significantly by using 3D6-Fab combined with PM system compared with that of applying free 3D6-Fab (Xie et al., 2020).

### The Combination of VHH and Liposomes

Liposomes are also used as carriers widely. Liposome contains an aqueous core and the lipid bilayer, whose unique structure is able to carry many different kinds of agents (Gopalan et al., 2020). Liposomes are nanometric and they can target certain domains specifically (Gopalan et al., 2020). VHH could be applied in the detection of Aβ deposits based on their high affinity to Aβ deposits. VHHs were also able to cross the model of BBB *in vitro*. VHHs were non-immunogenic and had been applied in clinical trials of human (Rotman et al., 2015a). According to the previous study, the renal clearance of VHH given in the intravenous way was rapid and the half-lives in blood were about 10–20 min, leading to the limited BBB passage. Therefore, GSH-PEG liposomes were developed in order to extend its time of staying in the blood, deliver VHH and make it pass through the BBB (Rotman et al., 2015a). This study offered a potential platform for diagnosing and treating AD by using VHH. VHH-pa2H was radiolabeled with indium-111 (^111^In) and DTPA so that VHH-pa2H itself could be traced (Rotman et al., 2015a). Two formulations of GSH-PEG liposomes which loaded VHH-pa2H-DTPA-^111^In were designed in the delivery of VHH. They found that liposomes were able to improve bioavailability and extend residence of VHH in the blood. In comparison with free VHH-pa2H, standard uptake values (SUV) were increased in the both formulations of GSH-PEG liposomes. VHH were able to be delivered into the brains of transgenic mice by GSH-PEG liposomes (Rotman et al., 2015a).

### The Combination of Antibody Fragments and Gold Nanoparticles

Gold nanoparticles (AuNPs) have excellent biocompatibility, stability and optical properties (Liu et al., 2019). Gold nanoparticles can be bioconjugated and functionalized easily. Their advantages make them become outstanding candidate in comparison with other nanoparticles. It was reported that combination of amyloid-degrading enzymes (ADEs), nanoparticles and scFvs had the capacity of reducing neurotoxicity by disaggregating Aβ fibrils and restraining aggregation of Aβ. Liu et al. (2019) made a complex called GNRs-APH-scFv (GAS). It was consisted of scFv 12B4, thermophilic acylpeptide hydrolase (APH) ST0779 and gold nanorods (GNRs). GNRs could produce hyperthermia by converting optical energy. GNRs were more effective in absorbing energy of near infrared (NIR) light than spherical AuNPs (Liu et al., 2019). Local hyperthermia could dissolve Aβ aggregations. ScFv binding to Aβ oligomers and fibrils, made hyperthermia specific, which decreased damage to other normal tissues. ST0779 could hydrolyze Aβ monomers. Multifunctional GAS complex could detect aggregation of Aβ and clear excessive Aβ by activating this entire system with NIR light after detecting Aβ. This complex can restrain aggregation of Aβ, disassemble fibrils of Aβ and decrease activity of peroxidase caused by Aβ. The Aβ cytotoxicity was decreased (Liu et al., 2019). Besides, there is another study about AuNPs and antibody fragments. An antibody against fibril of A*β*_1-42_ is called OC. Immobilizing the half fragment of OC on AuNPs was able to make an immunosensor. Palla et al. (2021) proved that the immunosensor could detect fibril of Aβ _1-42_ and evaluate the progress of AD (Palla et al., 2021).

### The Combination of Antibody Fragments and Superparamagnetic Iron Oxide Nanoparticles

Superparamagnetic iron oxide nanoparticles (SPIONs) are usually used in imaging as MRI contrast agents (Thangudu et al., 2020). Liu X.-G. et al. (2020) made a system called W20/XD4-SPIONs. They studied the effects of it on diagnosing and treating AD. SPIONs can transport through the BBB because of their small sizes. Aβ oligomers (AβOs) have important effect on causing dementia. W20, which is a scFv, could identify oligomers of amyloid specifically (Liu X.-G. et al., 2020). Phagocytosis of AβOs could be boosted by XD4. The class A scavenger receptor (SR-A) was activated by XD4. PEGylated SPIONs were combined with XD4 and W20. It was studied that W20/XD4-SPIONs boosted the phagocytosis of Aβ obviously because of the effect of XD40 on SR-A. W20/XD4-SPIONs could pass through the BBB. They could arrive at region of oligomers in the brain of AD transgenic mice according to the MRI. Their combinations with AβOs were sensitive and specific. They can be used in the diagnosis of AD in the early phase. Besides, W20/XD4-SPIONs could be used in treating AD because they can restrain the aggregation of Aβ. (Liu X.-G. et al., 2020). It was proved that W20/XD4-SPIONs could reduce cytotoxicity caused by AβOs. As for microglia, phagocytosis of Aβ was improved. And the cognition impairments and neuropathology were ameliorated by W20/XD4-SPIONs in AD mice (Liu X.-g. et al., 2020).

Taken together, different nanoparticles can be combined with antibody fragments including scFv, Fab fragment and single-domain antibody fragment. Their combination could facilitate the penetration of BBB and provide multiple therapeutic effects. Among these complexes, we prefer the combined application of antibody fragments and gold nanoparticles, which integrated diagnosis and treatment in one system. And local hyperthermia could dissolve Aβ aggregations. Furthermore, multifunctional GAS complex had less side effects due to its high specificity. Therefore, the combination of nanoparticles and antibody fragments may have more advantages in diagnosing and treating AD.

## Summary

It is promising to apply antibody fragments against Aβ in the diagnosis and treatment of AD. Antibody fragments have been further developed, because of their advantages over mAbs. Nanoparticles are potential candidates for drug delivery. A very important advantage of nanoparticles is that they can bind with many substances and deliver them to targeted regions. Nanoparticles can bind different agents with multiple functions to treat AD, which may take synergistic effects. Besides, nanoparticles can be combined with detective agents and treatment agents at the same time, integrating diagnosis and treatment in one system. Therefore, the combination of antibody fragments and nanoparticles becomes a system. This system can preserve and even enhance the functions of antibody fragments. In addition, many aspects should be paid attention to and further studied, for example, improving the specificity and stability of the combination and evaluating the potential toxicity. In the future, more and more combined application of antibody fragments and nanoparticles will be developed. All in all, combining antibody fragments with nanoparticles is a promising strategy in the diagnosis and treatment of AD.

## Glossary

AD: Alzheimer’s disease
Aβ: amyloid-β
AAV: adeno-associated virus
AβO: Aβ oligomer
ADE: amyloid-degrading enzyme
APH: acylpeptide hydrolase
BBB: blood-brain barrier
BSA: bovine serum albumin
CDR: complementarity-determining region
CSF: cerebrospinal fluid
CPC: chitosan polymeric core
CNV: control nano-vehicle
CYC: cyclophosphamide
CAA: cerebral amyloid angiopathy
DCN: deep cerebellar nuclei
Fc: crystallizable fraction
GAS: GNRs-APH-scFv
GNR: gold nanorod
GAβ: ganglioside-bound Aβ
MWM: Morris Water Maze
MP: mimetic peptide
MW: molecular-weight
mAb: monoclonal antibody
NIR: near infrared
PM: polymeric nanomicelle
rAAV: recombinant adeno-associated virus
scFv: single chain variable fragment
SNV: smart nano-vehicle
SUV: standard uptake values
SPION: superparamagnetic iron oxide nanoparticle
SR-A: class A scavenger receptor
TNV: theranostic nanovehicle
